# From Digital Anxiety to Empowerment in Older Adults: Cross-Sectional Survey Study on Psychosocial Drivers of Digital Literacy

**DOI:** 10.2196/75245

**Published:** 2026-01-21

**Authors:** Han-Jen Niu, Ming-Hsuan Li, Feng-Yu Hsieh, Chun-Chieh Yu, Chun-Ting Lin

**Affiliations:** 1 Management Science, College of Business and Management, Tamkang University, No. 151 Ying-Chung Rd., New Taipei City, 25137, Taiwan, 886 26215656 ext 2695; 2 Physical Education Instruction and Activities Section, Office of Physical Education, Tamkang University, New Taipei City, 25137, Taiwan; 3 AgriSense Agri-Food Education Association, New Taipei City, Taiwan

**Keywords:** digital literacy, successful aging, family support, social influence, technology acceptance, older adults, psychological moderation, digital anxiety

## Abstract

**Background:**

Amid the convergence of global population aging and accelerating digital transformation, older adults’ digital adaptability has emerged as a critical indicator of their quality of life, autonomy, and capacity for successful aging. However, digital disparities, technology-related anxiety, and insufficient support systems continue to hinder older individuals from fully participating in digital society. Particularly in modern family structures—where children often live apart from aging parents—the diminishing role of family support further underscores the importance of broader social influences.

**Objective:**

This study aims to examine how environmental factors (family support and social influence) and psychological factors (digital anxiety and sense of achievement) are associated with older adults’ intention to use Assistive Digital Tools and Services (ADTS), and how these relationships contribute to the development of digital literacy. Drawing upon an integrative framework that combines constructs from the Technology Acceptance Model, the Unified Theory of Acceptance and Use of Technology, and social cognitive theory, the study also investigates the mediating and moderating mechanisms underlying these effects, offering strategic insights to support older adults in moving from social isolation to digital empowerment.

**Methods:**

A structured questionnaire survey was conducted using a convenience sampling method among adults aged 55 years and older in Shenyang, Liaoning Province, China, yielding 480 valid responses. Structural equation modeling, bootstrapping, and moderation analysis were used to test the proposed integrative framework.

**Results:**

For both family support and social influence, their associations with digital literacy were fully mediated by ADTS. Higher family support was associated with lower digital anxiety, which in turn correlated with greater intention, while stronger social influence was directly associated with higher intention. Digital anxiety showed a strong negative association with intention; however, this relationship was significantly weaker among those reporting a higher sense of achievement. These findings highlight usage intention as a central pathway through which environmental and psychological conditions are related to digital competence.

**Conclusions:**

Digital literacy in later life is more than a technical skill set—it represents a vital form of psychological and social capital that empowers autonomy, well-being, and social integration. Strengthening older adults’ intention to engage with digital tools through emotional reinforcement, achievement-oriented experiences, and supportive social environments is key to narrowing the digital divide. Beyond its personal benefits, fostering digital competence contributes to successful aging, which in turn brings profound advantages for families, strengthens community cohesion, and supports national goals in public health, economic participation, and social sustainability. Intergenerational learning initiatives, community-based engagement programs, and leveraging social influence to offset weakened family support can create a more inclusive, resilient, and age-friendly digital ecosystem—one that benefits not only older individuals but society at large.

## Introduction

### Global Aging and the Challenge of Digital Inclusion

The intersection of rapid global population aging and accelerated digital transformation has rendered digital inclusion among older adults a pressing concern for researchers and policymakers alike. According to the United Nations [1], by 2050, the global population aged 65 years and older is projected to surpass 1.6 billion, accounting for nearly 16% of the total population. This demographic shift not only places strain on health care systems and labor markets but also redefines the societal roles and needs of aging populations in an increasingly digital world.

Within this context, digital literacy—encompassing not only operational skills but also digital confidence and information navigation—has been widely recognized as a key enabler for bridging this divide and promoting the effective use of Assistive Digital Tools and Services (ADTS) [2].

Prior studies have linked digital literacy to improved psychological well-being, life satisfaction, and learning motivation among older adults [3]. Yet, how digital literacy develops under the influence of environmental and psychological factors—especially in the context of aging—remains underexplored.

Digital exclusion exacerbates social isolation and loneliness among older adults, increasing the risk of depression and cognitive decline—while digital literacy offers a potential buffer against these outcomes [4]. In digitally mediated societies, older adults may experience compounded vulnerability—excluded not only socially but technologically.

Crucially, digital literacy holds promise as a remedy to both social and digital isolation. Older adults with strong digital competencies are more likely to engage in video communication, access health services remotely, and maintain active social networks [5,6]. In this sense, digital literacy is not merely a technical skill—it is a bridge to successful aging.

### COVID-19: A Double-Edged Catalyst for Digital Transformation

The COVID-19 pandemic served as a global stress test for digital readiness, rapidly shifting key aspects of daily life—health care, communication, commerce—into digital spaces. While digital services such as telemedicine and online grocery delivery became lifelines for many, they also exposed and widened the digital gap among older populations [7].

Older adults demonstrated significantly lower intention to adopt digital services during and after the pandemic, largely due to digital anxiety, low confidence, and lack of digital trust [8]. Digital anxiety encompasses fears about making mistakes, information overload, and concerns about fraud and data breaches—factors particularly salient for older first-time users [9].

While digital literacy training can alleviate anxiety and improve adoption, sustained engagement often depends on ongoing emotional and environmental support—particularly from family and community contexts [10]. However, the long-term sustainability of digital engagement often depends on external support structures such as family guidance or community-based learning. Without sustained environmental and emotional support, even trained users may regress into avoidance behaviors. This underscores the importance of examining how family and social influences shape not only technology adoption but also digital confidence and persistence among older adults.

### Research Gaps and Objectives

While widely adopted models such as the Technology Acceptance Model (TAM) and the Unified Theory of Acceptance and Use of Technology (UTAUT) have contributed significantly to understanding digital behavior, they were primarily developed for younger, working-age users [11]. As such, these models often overlook emotional vulnerabilities and contextual factors that are particularly salient among older adults—such as digital anxiety, family support, and social influence.

At the same time, the Digital Literacy Framework highlights competencies essential for navigating the digital world but does not explicitly address how these skills develop within psychosocial environments. Moreover, existing studies tend to examine these psychological and environmental variables in isolation, without a cohesive explanatory structure [5,11].

To address this gap, we propose an integrative framework that draws on TAM, UTAUT, and social cognitive theory (SCT) [12]. In this model:

- Family support and social influence reflect environmental enablers of technology adoption (UTAUT);
- Digital anxiety and sense of achievement represent emotional and cognitive mechanisms (SCT);
- Usage intention (from TAM) serves as a behavioral mediator;
- Digital literacy is positioned as a dynamic outcome representing both skill and empowerment.

Furthermore, we incorporate a moderated mediation structure, positing that sense of achievement may buffer the negative impact of digital anxiety on usage intention, thereby shaping digital literacy development [12].

This integrative approach also aligns with Successful Aging Theory, framing digital literacy as a form of “aging capital” that enhances autonomy, social participation, and psychological well-being.

Based on this framework, our study addresses the following research questions:

1. To what extent are family support and social influence associated with older adults’ intention to adopt ADTS?
2. Does digital anxiety negatively affect usage intention, and is this effect moderated by sense of achievement?
3. Does usage intention mediate the relationship between environmental/psychological factors and digital literacy?

By empirically validating this multilevel framework, we aim to offer new insights into how older adults move from digital exclusion to empowerment in an aging digital society.

In applying UTAUT as a guiding framework, we intentionally retained social influence and conceptualized family support as an environmental enabler, while excluding performance expectancy and effort expectancy. We do so for parsimony and contextual salience: (1) their predictive roles have been robustly established in prior work, so retesting them would add replication rather than novel insight; (2) prestudy interviews suggested many older adults lacked sufficient hands-on ADTS experience to meaningfully assess usefulness or ease of use; and (3) our theoretical focus is the age-specific psychosocial mechanisms (family support, social influence, anxiety, and achievement) that drive intention in later life. This selective adaptation contextualizes UTAUT rather than diluting it.

We also model digital literacy as an outcome—an accumulated form of “aging capital”—to trace how environmental and psychological conditions translate into competence via usage intention. While prior studies have treated literacy as an antecedent that can reduce anxiety and strengthen intention, our outcome-focused specification clarifies the developmental pathway we test here; it does not preclude reciprocal dynamics, which we note as an avenue for longitudinal research.

### Literature Review

#### Family Support

Family support plays a critical role in facilitating older adults’ digital engagement. Defined broadly, family support encompasses emotional encouragement, technical guidance, and intergenerational interaction that collectively enhance older adults’ adaptation to digital life [13]. Caplan [14] viewed the family as a key provider of values and behavioral norms, offering essential mediation when individuals encounter challenges. Casper et al [15] further divided family support into instrumental, financial, and emotional dimensions, all of which contribute meaningfully to older adults’ ability to learn and apply new technologies.

In the digital context, family members frequently act as facilitators in older adults’ learning process, offering real-time help and reassurance [16]. Older adults are often influenced by their children’s or grandchildren’s attitudes toward technology, making family encouragement a significant determinant of their intention to engage with ADTS [17].

Empirical research supports this relationship. Xiong and Zuo [13] showed that emotional support and technical instruction from family members enhance digital literacy by reducing fear and uncertainty. Similarly, Meng et al [18] and Sosa Díaz [19] found that positive intergenerational communication increases digital confidence and learning motivation. Sosa Díaz [19] highlighted that assistance from younger family members strengthens adaptability, while Roman et al [20] emphasized how strong family bonds can boost learning confidence.

Thus, this study highlights family support as a foundational environmental factor influencing both psychological readiness and behavioral engagement in digital contexts. The focus on intergenerational interaction aims to deepen our understanding of how familial dynamics contribute to digital inclusion among older adults.

#### Social Influence

Social influence refers to the degree to which individuals’ behaviors, attitudes, or decisions are shaped by those in their social environment—such as family, friends, or peers [11]. Rooted in the UTAUT, social influence is recognized as a key driver of technology adoption, particularly through normative pressure and perceived expectations from significant others [11,21].

From a broader psychological perspective, social influence encompasses beliefs, emotions, and behavioral patterns formed through interactions within social networks [21]. Morosan et al [22] highlighted that individuals’ perceptions of others’ expectations can significantly affect their own technology acceptance decisions. Karahanna et al [21] expanded this view by discussing how proximity, contact frequency, and interpersonal dynamics influence behavior, emphasizing that social structures can either facilitate or inhibit digital engagement.

Among older adults, social influence is particularly potent. Compared with younger individuals, older adults are more likely to rely on interpersonal cues and social norms when evaluating new technologies [11]. The impact of social influence is also more pronounced in collectivist cultures—such as in many East Asian societies—where conformity to group norms and maintaining social harmony are especially valued.

The role of social influence has become even more salient in the postpandemic digital era. During COVID-19, social distancing measures drove many older adults to adopt digital tools for health, communication, and daily tasks, often under the encouragement or guidance of their social circles [23]. Positive social reinforcement can lead to greater confidence and higher willingness to engage with digital services, whereas skepticism or lack of support may result in avoidance or anxiety [24-26].

Furthermore, recent studies show that social influence not only affects direct behavioral intention but also moderates psychological factors such as digital anxiety and self-efficacy [27]. These findings underscore the need to understand social influence as both an external motivator and a psychological buffer or amplifier in older adults’ digital adaptation.

By focusing on the multifaceted nature of social influence—including social norms, peer encouragement, and perceived expectations—this study aims to clarify how social context shapes older adults’ engagement with ADTS and the broader development of digital literacy.

### Digital Anxiety: Concept and Development

Digital anxiety refers to the emotional discomfort, fear, or stress that individuals—particularly older adults—experience when interacting with digital technologies. Unlike simple unfamiliarity, digital anxiety reflects a deeper psychological resistance often rooted in low confidence, fear of failure, and perceptions of complexity or risk [26,28]. Wang and Zhang [29] defined it as a form of state anxiety, varying with task demands and context. Among older adults, this anxiety is especially pronounced due to the widening digital divide and their limited prior exposure to emerging technologies.

Rook [30] highlighted that older individuals frequently report elevated anxiety when using ADTSs such as telemedicine platforms, smartphones, or mobile payments. This is compounded when they lack the digital self-efficacy or training needed to operate these tools confidently. Straub [26] found that prior experience and perceived self-efficacy can significantly reduce anxiety and increase willingness to adopt digital tools. Similarly, the TAM posits that perceived ease of use and usefulness directly shape user attitudes and behaviors.

From the perspective of Rogers’ Diffusion of Innovations Theory, perceived complexity and compatibility with past experiences are key barriers for older adults. Even simple digital interfaces can feel cognitively taxing, potentially triggering avoidance behaviors rather than active engagement [31,32].

One of the most consistently supported buffers against digital anxiety is family support. Beyond emotional reassurance, family members often provide practical technical guidance and encouragement, helping older adults feel less overwhelmed and more motivated to engage with digital tools. Studies have shown that such support fosters emotional security, strengthens digital self-confidence, and decreases feelings of uncertainty in digital environments [33]. For instance, when family members offer direct assistance—such as walking through digital tasks step-by-step—older adults are more likely to persist in their learning process and overcome initial apprehension.

However, overly reliant support can have mixed effects. While moderate, empowering support improves outcomes, excessive dependence may inadvertently signal incompetence or fuel learned helplessness, thereby reinforcing anxiety [7,34]. Nevertheless, the preponderance of evidence suggests a net protective role of family involvement in alleviating digital stress, leading to the following hypothesis:

 H1: Family support negatively influences digital anxiety.

In addition to family, broader social influence plays a crucial role in shaping digital anxiety. When older adults perceive positive expectations or encouragement from peers, neighbors, or community members, it can reduce fear and promote digital exploration. On the contrary, negative social feedback—such as expressions of doubt, impatience, or age-related stereotypes—can heighten self-doubt and anxiety. Recent studies have demonstrated that social environments that are judgmental or unsupportive intensify older adults’ digital apprehension, especially when they fear being seen as “incompetent” or “too old to learn” [35,36].

Conversely, positive social modeling and group-based learning environments—such as community digital workshops—can boost older adults’ digital confidence and reduce anxiety levels. Venkatesh et al [11] and Cambre and Cook [27] have also pointed out that perceived social expectations (a key dimension of UTAUT) can influence both emotional responses and behavioral intentions related to technology.

 H2: Social influence positively influences digital anxiety.

In summary, digital anxiety among older adults is shaped not only by individual cognitive appraisals but also by the presence—or absence—of emotional and environmental support. Understanding how family support and social influence interact with psychological states like anxiety is essential for building more inclusive and effective digital interventions.

### Usage Intention of Assistive Digital Tools and Services

The intention to use ADTS among older adults is a central construct in models of technology adoption. Usage intention refers to an individual’s motivational readiness and willingness to adopt or engage with a specific technology [37]. In the context of aging populations, intention plays a critical intermediary role, linking psychological and environmental factors with actual digital behavior and literacy development.

### Family Support and Usage Intention

Family support has been widely documented as a catalyst for technology acceptance among older adults. Beyond alleviating anxiety, supportive family environments contribute to a more proactive stance toward digital learning. Emotional encouragement, technical guidance, and shared experiences with family members strengthen older adults’ belief in their own capability, thereby enhancing their readiness to use ADTS.

Studies suggest that family members who model digital behavior or provide hands-on help not only increase access but also shape attitudes of usefulness and ease of use—2 key predictors in the TAM [38]. For instance, Zhang [17] found that the opinions of close family members significantly influenced older adults’ willingness to try new technologies. Likewise, Selwyn [16] and Park [39] noted that active family involvement enhances digital confidence and curiosity, which translates into a stronger intention to use tools like mobile banking or telemedicine services.

While over-dependence on family for digital engagement may hinder independent learning, moderate and empowering support appears to foster technology acceptance. Therefore, based on the strong empirical and theoretical link between family support and technology adoption, the following hypothesis is proposed:

 H3: Family support positively influences older adults’ intention to use ADTS.

### Social Influence and Usage Intention

Social influence extends beyond the family unit to include peers, community members, and broader societal norms that shape older adults’ willingness to engage with digital technologies. In UTAUT, social influence is considered a direct antecedent of usage intention, reflecting how individuals perceive the expectations and behaviors of others as relevant to their own decision-making [11].

Research demonstrates that when older adults observe peers successfully using ADTS, they are more likely to emulate those behaviors, especially in collectivist cultures where conformity and social approval are emphasized [40,41]. Additionally, informational and normative forms of social influence—such as digital learning groups, community workshops, or word-of-mouth recommendations—play a crucial role in shaping perceived accessibility and relevance of digital services [42,43].

Moreover, positive social reinforcement enhances self-efficacy, reduces uncertainty, and increases perceived behavioral control—factors that strongly predict technology use. Conversely, negative perceptions within one’s social network may lead to skepticism or hesitation. Given this evidence, we propose:

 H4: Social influence positively influences older adults’ intention to use ADTS.

### Digital Anxiety and Usage Intention

Digital anxiety, as established in prior sections, serves as a psychological inhibitor that can undermine willingness to engage with ADTS. Fear of making mistakes, concerns over privacy, and low digital confidence create a barrier that suppresses both motivation and perceived ability.

Drawing from TAM and SCT, when anxiety increases, perceived ease of use diminishes, leading to lower adoption intention [3,9]. In real-world contexts, this means older adults who feel overwhelmed or unsupported are more likely to avoid digital tools, even when such tools offer substantial benefits.

Studies in health care technology and fintech show consistent negative correlations between digital anxiety and usage intention [44,45]. Lee et al [9] emphasized that enhancing digital self-efficacy can reduce anxiety and indirectly boost usage intention, but when anxiety remains unaddressed, intention significantly declines. Given this robust evidence, we hypothesize:

 H5: Digital anxiety negatively influences older adults’ intention to use ADTS.

### Digital Literacy

Digital literacy is a multidimensional competency that encompasses not only the ability to use digital tools, but also to evaluate, create, and communicate digital content effectively [46,47]. It includes technical, cognitive, and socioemotional skills, all of which are crucial for navigating digital environments [48,49]. In aging populations, digital literacy has emerged as a key determinant of autonomy, participation, and well-being [50].

Research has shown that the intention to use digital tools often precedes and fosters the development of digital literacy. When older adults are motivated to use digital tools—such as smartphones, e-payment systems, or telehealth platforms—they are more likely to develop the necessary skills and confidence through experiential learning [51,52]. This iterative process suggests that usage intention is not merely an outcome but also a catalyst in digital literacy acquisition.

 H6: Usage intention of ADTS positively influences digital literacy.

Beyond direct effects, usage intention also serves as a mediating mechanism through which environmental support influences digital literacy. For instance, when family members provide technical guidance and emotional encouragement, they boost older adults’ confidence and motivation. This, in turn, leads to increased digital usage, which fosters skill development and digital empowerment [38,53].

 H7: Usage intention of ADTS mediates the relationship between family support and digital literacy.

Similarly, social influence plays a significant role in digital skill development. Older adults who are embedded in digitally engaged social networks are more likely to receive informal training, tips, and positive reinforcement, which enhance both their usage intention and their digital competence [35,41,42]. This cascading effect suggests that social influence indirectly contributes to digital literacy via behavioral intention.

 H8: Usage intention of ADTS mediates the relationship between social influence and digital literacy.

### Sense of Achievement

Sense of achievement refers to the intrinsic satisfaction and psychological reward individuals experience upon successfully accomplishing a task, overcoming a challenge, or attaining a self-defined goal [54,55]. This construct is closely tied to self-efficacy, motivation, and emotional resilience—factors that are especially relevant when individuals are navigating unfamiliar or cognitively demanding environments.

For older adults adapting to digital life, a sense of achievement plays a pivotal role. Unlike younger users who often grow up immersed in digital environments, older adults frequently approach technology as late adopters. When they successfully use digital tools—such as conducting a video call, managing a mobile payment, or accessing web-based health services—this sense of accomplishment becomes a powerful motivator for further learning and sustained engagement [56]. It also fosters autonomy and contributes to their psychological well-being, aligning with key principles of successful aging.

However, the digital environment presents numerous barriers that can threaten or diminish this feeling of accomplishment. These include perceived complexity, lack of intuitive design, cybersecurity concerns, and insufficient support systems [57]. When older adults encounter repeated failure or confusion, their confidence may erode, reinforcing negative beliefs about their ability to master technology. In this context, a diminished sense of achievement can magnify digital anxiety and lead to avoidance behaviors.

Recent studies suggest that a strong sense of achievement can buffer the negative effects of digital anxiety. Older adults who perceive themselves as capable learners are more likely to persist through technological challenges and less likely to internalize failure as a reflection of their overall competence [9]. In contrast, those who lack this sense of accomplishment are more prone to technostress and self-doubt—factors that reduce usage intention [58].

Given these dynamics, this study positions sense of achievement as a moderator in the relationship between digital anxiety and the intention to use ADTS. Specifically, we propose that achievement-oriented individuals will experience a weaker negative effect of digital anxiety on usage intention, as their internal sense of progress and mastery helps to offset fear and hesitation.

 H9: Sense of achievement moderates the relationship between digital anxiety and usage intention of ADTS, such that the negative effect is weaker at higher levels of achievement.

## Methods

### Participants

This study focuses on older adults in Shenyang, Liaoning Province, China, considering the region’s demographic aging trends and digital adaptation status. As one of the major cities in Northeast China, Shenyang has a rapidly aging population, with individuals aged 60 years and older accounting for over 20% of its residents, meeting the United Nations’ definition of a “deeply aging society”[96]. With the advancement of digital technologies, the internet behaviors and technological adaptation of older adults have become critical research topics. However, despite Shenyang’s well-developed urban infrastructure, older adults in the region still face significant challenges in using ADTS (smartphones and e-services, etc).

Previous research has highlighted the existence of a digital divide among China’s aging population, which is particularly pronounced in the northeastern region. Chu (2010) further pointed out that older individuals’ digital learning and technology adaptation are influenced by educational background, social support, and technology anxiety, all of which contribute to lower adoption rates of e-services. These factors may limit older adults’ ability to access web-based resources, subsequently affecting their digital quality of life [13].

The socioeconomic structure of Shenyang also plays a crucial role in shaping older adults’ digital behaviors. As a traditional industrial city, Shenyang has a significant population of retirees from state-owned enterprises, whose social support networks and digital behavior patterns differ from those in coastal cities or rural areas [5]. Research by Chen et al [5] has further demonstrated a correlation between older adults’ socioeconomic status, online activity types, and susceptibility to digital fraud, emphasizing the importance of digital literacy in ensuring their safety on the web. Given these factors, this study selects Shenyang as its research site to gain deeper insights into older adults’ behavioral patterns and psychological influences in digital environments.

Additionally, this study includes individuals aged 55 years and older rather than limiting the sample to traditional older adult groups. There are 2 key reasons for this decision. First, individuals in this age group are rapidly adopting internet technologies, making them a highly relevant demographic for this study. Second, early retirement is common in China, with many individuals experiencing retirement before the official retirement age. By incorporating participants aged 55 years and older, this study effectively captures the early stages of aging and the transition into digital adaptation [5].

This study used a convenience sampling method, with surveys distributed across community centers, senior activity venues, and elderly care institutions in Shenyang. Individuals aged 55 years and older were invited to voluntarily complete the questionnaire. A total of 541 responses were collected. After excluding 61 incomplete questionnaires, 480 valid responses were retained, resulting in an effective valid completion rate among respondents of 88.7%. All participants were residents of Shenyang, Liaoning Province, China.

**Figure 1.**
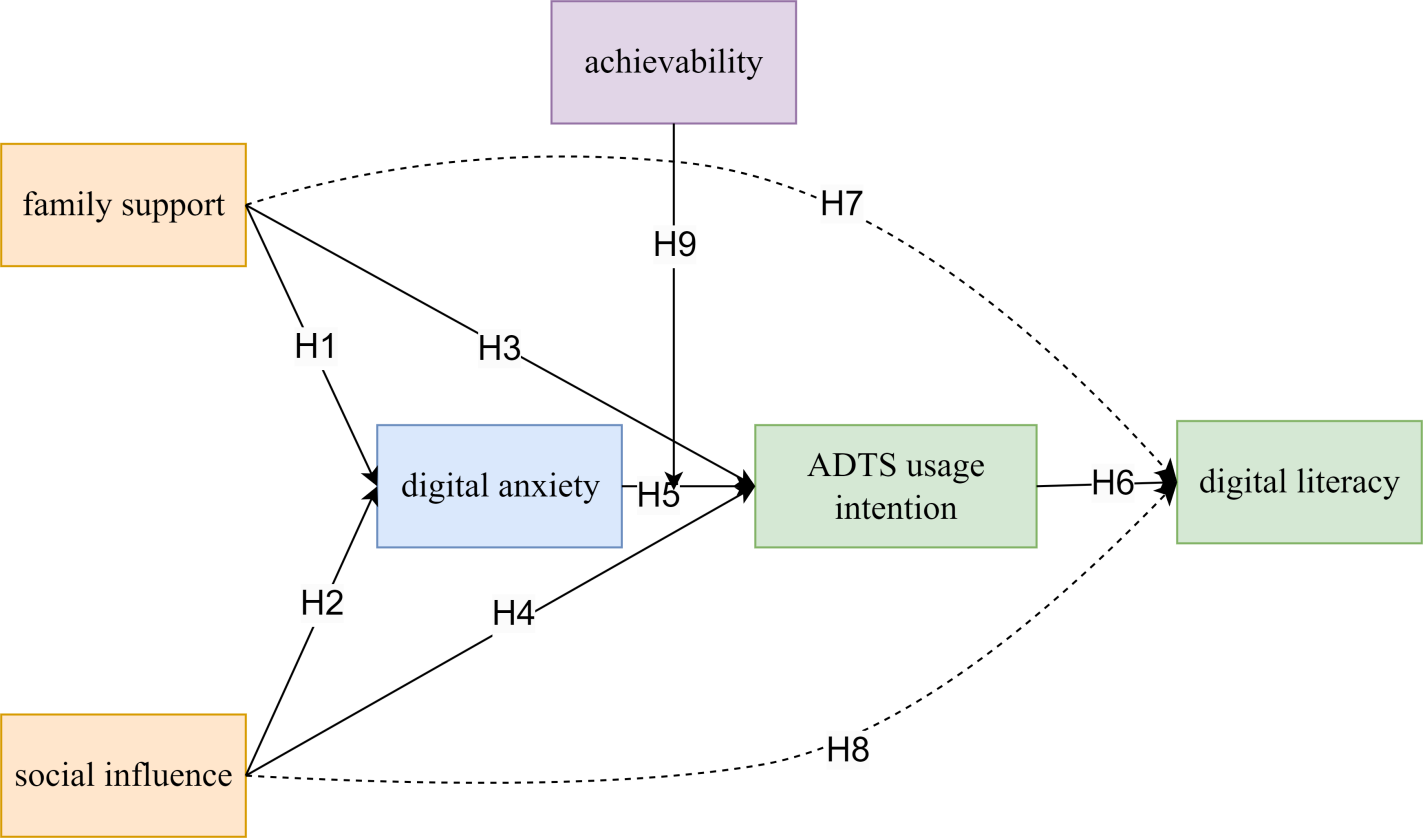
Research Framework. ADTS: Assistive Digital Tools and Services.

Demographic data were collected on participants’ gender, age, education level, marital status, family structure, place of residence, and smartphone usage behavior. To ensure accurate comprehension and response quality, the research team provided detailed instructions and on-site assistance during the survey process. The conceptual framework guiding this study is presented in Figure 1.

### Measures

This study primarily investigates the psychological states and digital behaviors of older adults. A 6-point Likert scale was used, ranging from “strongly disagree” to “strongly agree,” to measure key variables, including social influence, family support, digital anxiety, digital literacy, adoption intention of ADTS, and sense of achievement. The measurement instruments were derived from well-established scales in the existing literature:

Family support was adapted from Wang and Wu [28], originally developed in the context of older adults’ digital adaptation in urban China. The scale assesses 3 core dimensions of support provided by family members: emotional encouragement, technical assistance, and informational guidance. While Wang and Wu’s [28] study focused on digital literacy outcomes, the psychometric properties of the family support scale were independently validated through confirmatory factor analysis (CFA) and internal consistency measures (Cronbach *α*=0.82). This scale was chosen for its cultural contextual relevance and conceptual alignment with our study focus. In our sample, Cronbach α was 0.802.

Social influence was adapted from Venkatesh et al [11] in the UTAUT. The scale evaluates the extent to which individuals perceive that important others—such as family members, peers, or community figures—believe they should use digital technologies. Each item was rated on a 6-point Likert scale. This construct has been validated in numerous technology acceptance studies across diverse populations, including older adults. In this study, the scale demonstrated good internal consistency (Cronbach *α*=0.798).

Digital anxiety was also adapted from Venkatesh et al [11]. The items reflect the UTAUT construct of anxiety, which captures individuals’ apprehension, fear, or discomfort when interacting with digital technology. Although originally tested in younger user populations, the scale has been applied and adapted in aging research. In this study, internal consistency was acceptable (Cronbach *α*=0.762), supporting its reliability among older adult users.

Intention to use ADTS was adapted from the UTAUT model (Venkatesh et al [11]), assessing respondents’ willingness to engage with digital services such as e-health platforms, digital payments, and digital communication tools. Each item was rated on a 6-point Likert scale. The original scale has been validated in both general and older adult populations. In this study, Cronbach α was .789, indicating acceptable reliability.

Digital literacy was evaluated using the eHealth Literacy Scale developed by Norman and Skinner [59], consisting of 8 items that measure perceived ability to find, understand, evaluate, and apply digital health information. This scale has been widely adopted in aging, health, and digital divide studies and has demonstrated strong psychometric properties across diverse populations. In our study, the scale yielded a Cronbach α of 0.871, indicating high internal consistency.

Sense of achievement was measured using a 3-item subscale from the achievement motivation framework developed by Janke and Lüftenegger [50]. The items reflect individuals’ feelings of satisfaction and competence when overcoming digital challenges. The original instrument was validated in academic and motivational contexts and has since been adapted in studies of digital learning among older adults. The internal consistency for this scale in our sample was strong (Cronbach *α*=0.851).

To ensure the statistical adequacy of structural equation modeling (SEM), this study adhered to the sample size recommendations proposed by Bentler and Chou [60], which suggest that the sample size should be at least 5 times the number of estimated parameters. Additionally, data screening procedures, including missing data analysis and normality tests, were conducted to ensure the suitability of the dataset for subsequent analysis. Ultimately, a total of 480 valid responses were collected, meeting the theoretical requirements for robust statistical analysis.

Reliability (Cronbach α), CFA, and SEM were conducted using SPSS (version 26.0; IBM Corp) and AMOS (version 24.0; IBM Corp). Bootstrapping (n=5000) and PROCESS Macro (Model 1) were used to test mediation and moderation effects, ensuring robust statistical validity.

### Ethical Considerations

This study utilized fully anonymized survey data that contained no personally identifiable information. As such, ethical approval and informed consent were not required under institutional and national research ethics regulations. All research procedures adhered to internationally recognized ethical standards, ensuring transparency, data integrity, and the responsible handling of anonymized information.

## Results

### Sample Profile

Regarding gender distribution, 69% (n=331) of respondents were male, and 31% (n=149) were female. In terms of age, the majority were aged between 60 and 65 years (263/480, 54.8%), followed by those aged 65 to 70 years (167/480, 34.8%), while only 1.0% (n=5) were aged 75 years and older. Residential distribution indicated that 60.4% (n=290) of participants lived in urban areas, whereas 39.6% (n=190) resided in rural regions. In terms of educational attainment, 57.1% (n=274) of respondents had completed middle school or below, 23.5% (n=111) held a university degree, and 1.5% (n=7) had attained a postgraduate degree or higher. Regarding marital status, 87.5% (n=420) of respondents were married, 4.8% (n=23) were unmarried, 2.9% (n=14) were divorced, and 4.8% (n=23) were widowed. As for family structure, 74.8% (n=359) lived with their spouse, 12.5% (n=56) lived with their children, and 12.7% (n=61) lived alone.

Regarding the usage of ADTS, 85.0% (n=408) of respondents primarily used smartphones for voice calls, followed by text messaging (381/480, 79.4%) and video calls (357/480, 74.4%). In contrast, mobile shopping (261/480, 54.3%) and digital payments (271/480, 56.5%) were less frequently used. Notably, only 53.4% (n=256) of participants used their smartphones for digital information retrieval, suggesting that a considerable portion of older adults remains unfamiliar with internet-based search functions.

### Reliability Analysis

To assess the internal consistency of the measurement scales, this study calculated Cronbach α for each variable. The results, presented in Table 1, indicate that all Cronbach α values exceeded the recommended threshold of 0.7, demonstrating good reliability across the constructs.

**Table 1.** The results of reliability analysis (N=480). All constructs were measured on a 6-point Likert scale (1=strongly disagree, 6=strongly agree).

Variable	Values, mean (SD)	Values, Cronbach α
Social influence	2.850 (1.023)	0.798
Family support	4.960 (0.803)	0.802
Digital anxiety	4.980 (0.784)	0.762
ADTS^a^ usage intention	5.030 (0.844)	0.789
Digital literacy	4.510 (0.919)	0.871
Achievability	5.380 (0.817)	0.851

a ADTS: Assistive Digital Tools and Services.

Among the variables, Digital Literacy exhibited the highest internal consistency, with a Cronbach α of 0.871, suggesting strong reliability. Meanwhile, Digital Anxiety had a Cronbach α of 0.762, which remains within an acceptable range, indicating sufficient internal consistency for further analysis.

### Confirmatory Factor Analysis

To ensure convergent validity and discriminant validity of the measurement model, this study conducted CFA. Following the recommendations of Hair et al [61], composite reliability should exceed 0.7, and average variance extracted should be above 0.5 to confirm the internal consistency and validity of the measurement instruments.

As shown in Table 2, all constructs in this study met these criteria, with composite reliability values exceeding 0.7 and average variance extracted values above 0.5, indicating strong convergent validity. Additionally, all standardized factor loadings were statistically significant (>0.60), demonstrating that the observed variables effectively captured their respective latent constructs.

**Table 2.** Results of convergent validity analysis (N=480).

Concept and item	Standard factor loading	Composite reliability	Average variance extracted
Social influence (SI)		0.802	0.506
SI1	0.692		
SI3	0.818		
SI4	0.631		
F1	0.670		
Family support (F)		0.803	0.504
F3	0.705		
F4	0.748		
F5	0.716		
D2	0.586		
Digital literacy (D)		0.872	0.535
D4	0.690		
D5	0.696		
D6	0.808		
D7	0.798		
D8	0.785		
ADTS1^a^	0.765		
ADTS usage intention (ADTS**)**		0.802	0.578
ADTS2	0.865		
ADTS3	0.633		
DA1	0.704		
Digital anxiety (DA**)**		0.803	0.506
DA2	0.732		
DA3	0.729		
DA4	0.678		
A1	0.804		
Achievability (A)		0.880	0.711
A2	0.950		
A3	0.765		

a ADTS: Assistive Digital Tools and Services.

Overall, the results confirm that the measurement tools used in this study exhibit high reliability and validity, ensuring their suitability for further SEM analysis.

### Structural Equation Modeling (SEM) Analysis

#### Model Fit Assessment

**Figure 2.**
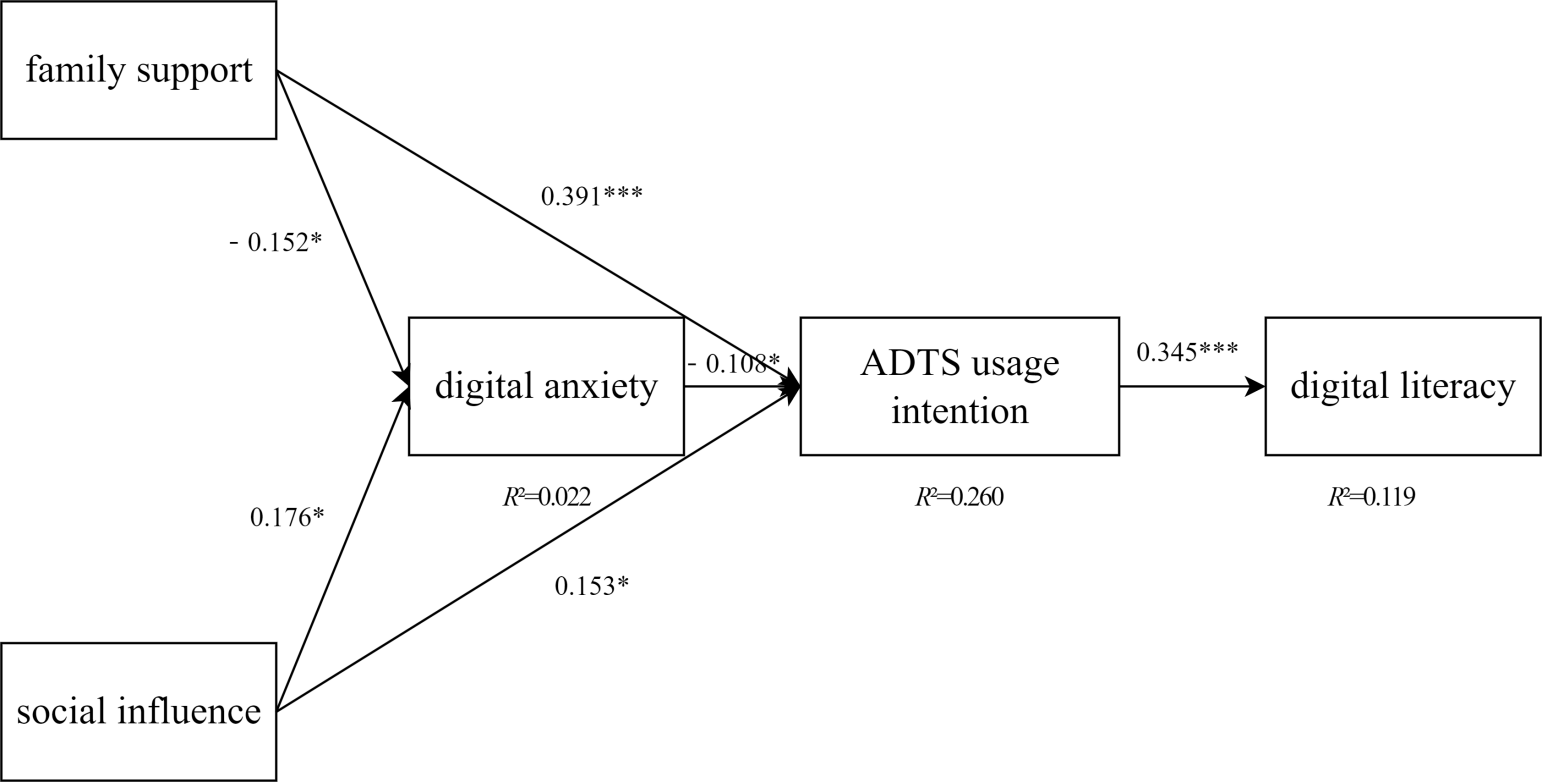
The model of structural equation modeling. ADTS: Assistive Digital Tools and Services. *P<.001, **P<.01, ***P<.05.

This study used SEM to validate the proposed research framework, as illustrated in Figure 2. Prior to conducting SEM analysis, we assessed the model fit indices to ensure the suitability of the model.

By examining the Modification Index, we identified and removed items with large residual discrepancies, including 2 items from the Family Support scale and items 1 and 3 from the Digital Literacy scale. After these modifications, the revised model exhibited improved goodness-of-fit indices, confirming its appropriateness for further SEM analysis (Table 3).

**Table 3.** Goodness of model fit indices (N=480).

Goodness of Fit Index	Scholars	Goodness fit range	Modified model (Delete SI2, D1, D3)
CMIN/DF^a^	Joreskog and Sorbom [52]	＜3.00	2.499
GFI^b^	Doll, Xia, and Torkzadeh [53]	＞0.8	0.925
AGFI^c^	MacCallum and Hong [62]	＞0.8	0.902
SRMR^d^	Hu and Bentler [63]	＜0.08	0.057
RMSEA^e^	MacCallum, Browne, and Sugawara [54]	＜0.08	0.053
NFI^f^	Hair et al [61]	＞0.8	0.902
NNFI^g^	Bentler and Bonett [55]	＞0.8	0.884
CFI^h^	Hair et al [61]	＞0.8	0.941
PNFI^i^	Mulaik et al [64]	＞0.5	0.751
PGFI^j^	Mulaik et al [64]	＞0.5	0.705
CN^k^	Mulaik et al [64]	＞200	259

a CMIN/DF: chi-square minimum/degrees of freedom.

b GFI: Goodness-of-Fit Index.

c AGFI: Adjusted Goodness-of-Fit Index.

d SRMR: Standardized Root Mean Square R.

e RMSEA: Root Mean Square Error of Approximation.

f NFI: Normed Fit Index.

g NNFI: Non-Normed Fit Index.

h CFI: Comparative Fit Index.

i PNFI: Parsimony Normed Fit Index.

j PGFI: Parsimony Goodness-of-Fit Index.

k CN: Hoelter’s Critical N.

#### Structural Equation Modeling (SEM) Analysis

The SEM analysis in this study was conducted in 2 stages. First, we examined the overall model fit indices to ensure the model’s suitability. Second, we tested the path coefficients to validate the research hypotheses.

By performing path analysis on the latent variables, we assessed the relationships among key constructs. The results are presented in Table 4 and Figure 2.

**Table 4.** The results of structural equation modeling path analysis.

Hypothesis	Independent variable	Dependent variable	β	SE	Composite reliability	*P* value
H1	F^a^	A^b^	−.152	0.080	–2.087	<.037
H2	SI^c^	A	.176	0.081	2.362	<.018
H3	F	ADTS^d^	.391	0.077	5.356	<.001
H4	SI	ADTS	.153	0.071	2.235	<.025
H5	A	ADTS	−.108	0.049	–2.112	<.035
H6	ADTS	D^e^	.345	0.041	6.031	<.001

a F: Family support.

b A: Achievability.

c SI: Social influence.

d ADTS: Assistive Digital Tools and Services.

e D: Digital literacy.

### Mediating Effect

This study used the PROCESS macro in SPSS (version 26.0; IBM Corp) with the bootstrap method to examine mediating effects. The analysis tested whether usage intention of ADTS mediated the relationships between (1) family support and digital literacy, and (2) social influence and digital literacy.

For the family support → digital literacy pathway (Table 5), the indirect effect was 0.071, with a 95% CI of (0.095-0.262), indicating statistical significance as the interval did not include zero. In contrast, the direct effect was 0.397, with a 95% CI of (–0.130 to 0.082), which was nonsignificant. This pattern reflects full mediation, meaning that family support influences digital literacy entirely through its impact on usage intention.

**Table 5.** Mediating effect analysis (family support and digital literacy).

F→ADTS→D^a^^,^^b^^,^^c^	Effect	Bootstrapping (BC^d^ 95% CI)
Indirect effect	0.071	0.095 to 0.262
Direct effect	0.397	–0.130 to 0.082
Total effect	0.468	0.003 to 0.052

a F: family support.

b ADTS: Assistive Digital Tools and Services.

c D: digital literacy.

d BC: bias-corrected percentile method.

For the social influence → digital literacy pathway (Table 6), the indirect effect was also significant (95% CI excluding zero), whereas the direct effect was −0.418 with a 95% CI of (−0.141 to 0.186), which was nonsignificant. This likewise indicates full mediation, suggesting that social influence affects digital literacy only indirectly via usage intention.

**Table 6.** Mediating effect analysis (social influence and digital literacy).

SI→ADTS→D^a^^,^^b^^,^^c^	Effect	Bootstrapping (BC^d^ 95% CI)
Indirect effect	0.128	0.069 to 0.194
Direct effect	–0.418	–0.141 to 0.186
Total effect	0.086	0.014 to 0.186

a SI: social influence.

b ADTS: Assistive Digital Tools and Services.

c D: digital literacy.

d BC: bias-corrected percentile method.

Overall, these findings demonstrate that both family support and social influence enhance older adults’ digital literacy not by directly improving their skills, but by strengthening their intention to use ADTS—thereby facilitating subsequent skill acquisition and competence development.

### Moderation Effect

The moderating effect of sense of achievement on the relationship between digital anxiety and ADTS usage intention was examined using PROCESS Model 1 with 5000 bootstrap samples. The overall model was significant (*R*^2^=0.389, *F*_1, 476_=101.222, *P*=.003), and the interaction term (Digital Anxiety×Sense of Achievement) was also significant, indicating the presence of a moderation effect.

**Figure 3.**
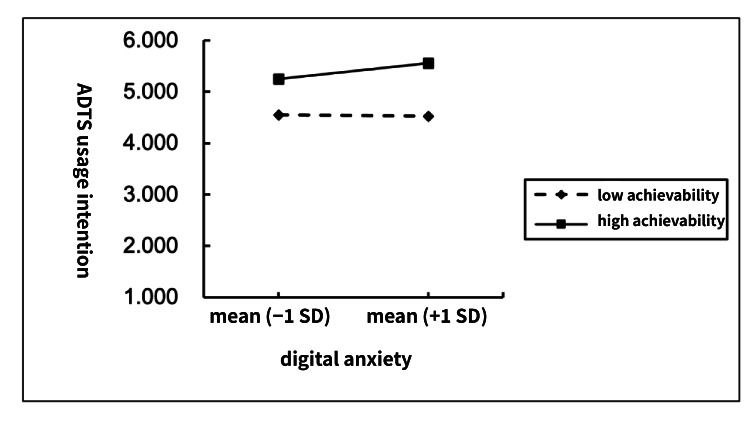
Moderation path coefficients. ADTS: Assistive Digital Tools and Services. *P<.05.

Simple-slopes analysis revealed that the negative association between digital anxiety and ADTS usage intention was weaker at higher levels of sense of achievement and stronger at lower levels. This suggests that a greater sense of achievement can buffer the detrimental impact of digital anxiety on the intention to use ADTS. Figures3  4 depict the slope differences across varying levels of sense of achievement, providing visual confirmation of the moderation effect.

In line with Self-Determination Theory, these results indicate that perceived competence and mastery help sustain motivation when engaging in tasks that may provoke anxiety. Accordingly, H9 is supported: sense of achievement attenuates the negative effect of digital anxiety on ADTS usage intention.

**Figure 4.**
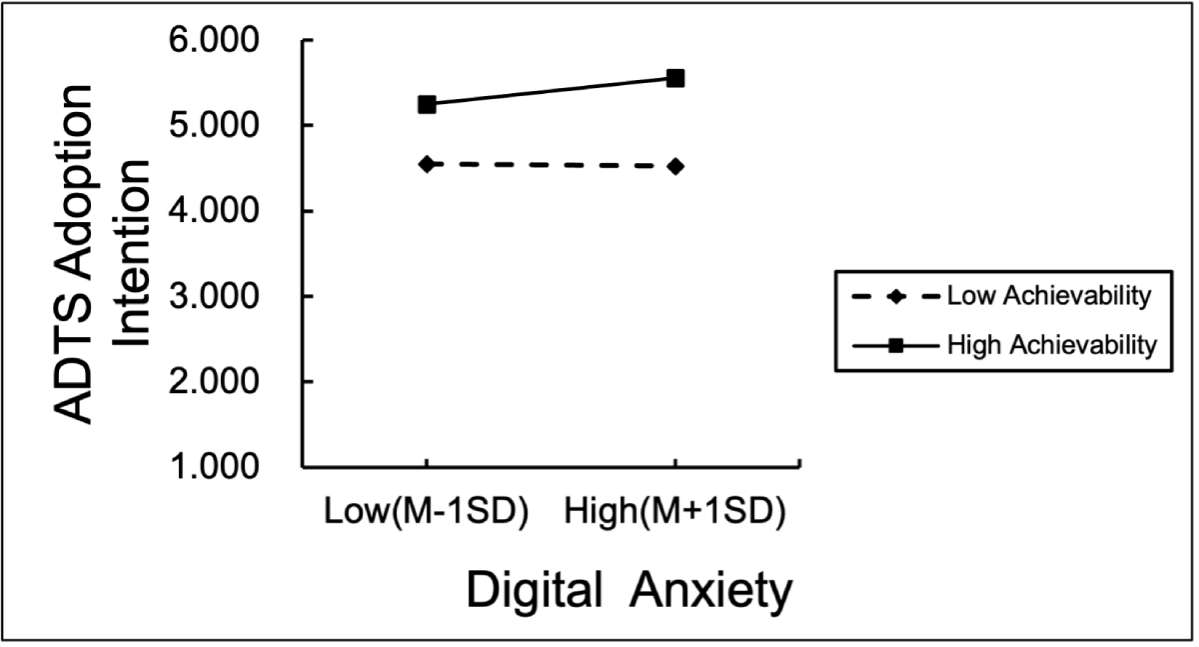
Moderation slope analysis. ADTS: Assistive Digital Tools and Services.

## Discussion

### Summary of Key Findings

In an era marked by rapid digital transformation and global population aging, older adults’ ability to adapt to digital technologies has become a critical determinant of quality of life, autonomy, and the trajectory of successful aging [2,3]. Grounded in the UTAUT and the Digital Literacy Framework, this cross-sectional study tested an integrated model that incorporated environmental, psychological, and behavioral factors. Specifically, it examined how family support, social influence, digital anxiety, and sense of achievement are associated with older adults’ intention to use ADTS, and how these factors, in turn, relate to the development of digital literacy. The findings provide new insights into how digital engagement can be transformed into real-life competence and social connection.

### Family Support and Social Influence Operate Through Intention

Both factors were positively associated with ADTS usage intention (*β*=.413 and *β*=.160, respectively, *P*<.05). Mediation analysis showed that their effects on digital literacy were fully mediated by usage intention, with nonsignificant direct paths after accounting for the mediator.

### Family Support Was Associated With Lower Digital Anxiety

Family support was negatively associated with digital anxiety (*β*=−0.168, *P*<.05), which in turn was negatively associated with usage intention (*β*=−.103, *P*<.05). This suggests that family support may help older adults overcome apprehension toward technology, indirectly promoting adoption.

### Sense of Achievement Attenuates the Association of the Negative Effect of Digital Anxiety

The interaction term (Digital Anxiety × Sense of Achievement) was significant, indicating that a higher sense of achievement attenuates the negative relationship between digital anxiety and usage intention.

### Usage Intention Is a Central Behavioral Pathway

Across environmental and psychological predictors, usage intention emerged as the pivotal link to higher digital literacy scores (*β*=.345, *P*<.001).

### Theoretical Implications

This study advances the literature by showing how psychosocial factors operate within an integrative, later-life adaptation of UTAUT. Specifically, it contributes three distinctive insights—each grounded in the SEM results:

1. Extending UTAUT with age-specific psychosocial mechanisms: Beyond utilitarian appraisals, digital anxiety exerts a negative effect on intention (β=−0.103, *P*<.05), while sense of achievement attenuates this link (significant interaction), indicating that affective constraints and motivational buffers are integral to older adults’ adoption processes.
2. Unpacking the pathway from environmental enablers to competence: The effects of family support (*β*=.413) and social influence (*β*=.160) on digital literacy are fully mediated by usage intention (indirect effects with 95% CIs excluding zero), positioning intention as a necessary conduit from context to competence.
3. Reframing successful aging for the digital era: Digital literacy functions as aging capital—a capability accumulated through intentional engagement and shaped by psychosocial conditions—thereby warranting its incorporation into the theoretical core of successful aging in technology-mediated societies.

### Practical / Managerial Implications

The findings translate into the following action priorities for policymakers, community organizations, and technology designers:

1. Leverage family and peer networks as complementary pillars: Given the stronger association of family support with intention (*β*=.413) relative to social influence (*β*=.160), intergenerational pairings can serve as high-impact entry points. To avoid over-dependence, pair these with peer-led workshops, senior tech clubs, and neighborhood “digital ambassadors” for continuity.
2. Design for progressive achievement to counter anxiety: Because achievement buffers the anxiety-intention link, use milestone-based modules, micro-goals (eg, a first mobile payment), and instant positive feedback to convert apprehension into mastery and sustained engagement.
3. Prioritize sustained, habitual use over one-off adoption: With literacy gains operating through intention, build reinforcement sessions, periodic “digital check-ins,” and adaptive nudges (reminders, personalized suggestions, calibrated difficulty) to routinize use and consolidate skills. These recommendations move beyond generic calls for support, advancing age-sensitive, culturally grounded strategies that directly target the psychosocial dynamics revealed by our model.

### Future Research and Practice Directions

#### Limitations and Future Directions

The cross-sectional design precludes causal inference; results reflect associations within one metropolitan sample, limiting generalizability. Demographic covariates (eg, age, education, marital status) were collected but excluded from the structural model to preserve parsimony; future work can incorporate them to refine estimates.

#### Modeling Choices

We intentionally excluded performance expectancy and effort expectancy to foreground later-life psychosocial mechanisms and avoid redundancy with well-established findings; future studies should reincorporate these UTAUT constructs to test robustness and generalizability. We also modeled digital literacy as an outcome to trace competence formation; longitudinal or cross-lagged designs should examine possible bidirectional effects whereby literacy reduces anxiety, enhances self-efficacy, and strengthens intention.

#### Next Steps

We recommend longitudinal/experimental designs, cross-cultural comparisons, and the inclusion of digital trust/privacy and emerging AI/IoT tools to evaluate scalable, age-friendly pathways to digital inclusion.

## Abbreviations

ADTS: Assistive Digital Tools and Services
CFA: confirmatory factor analysis
SCT: social cognitive theory
SEM: structural equation modeling
TAM: Technology Acceptance Model
UTAUT: Unified Theory of Acceptance and Use of Technology
